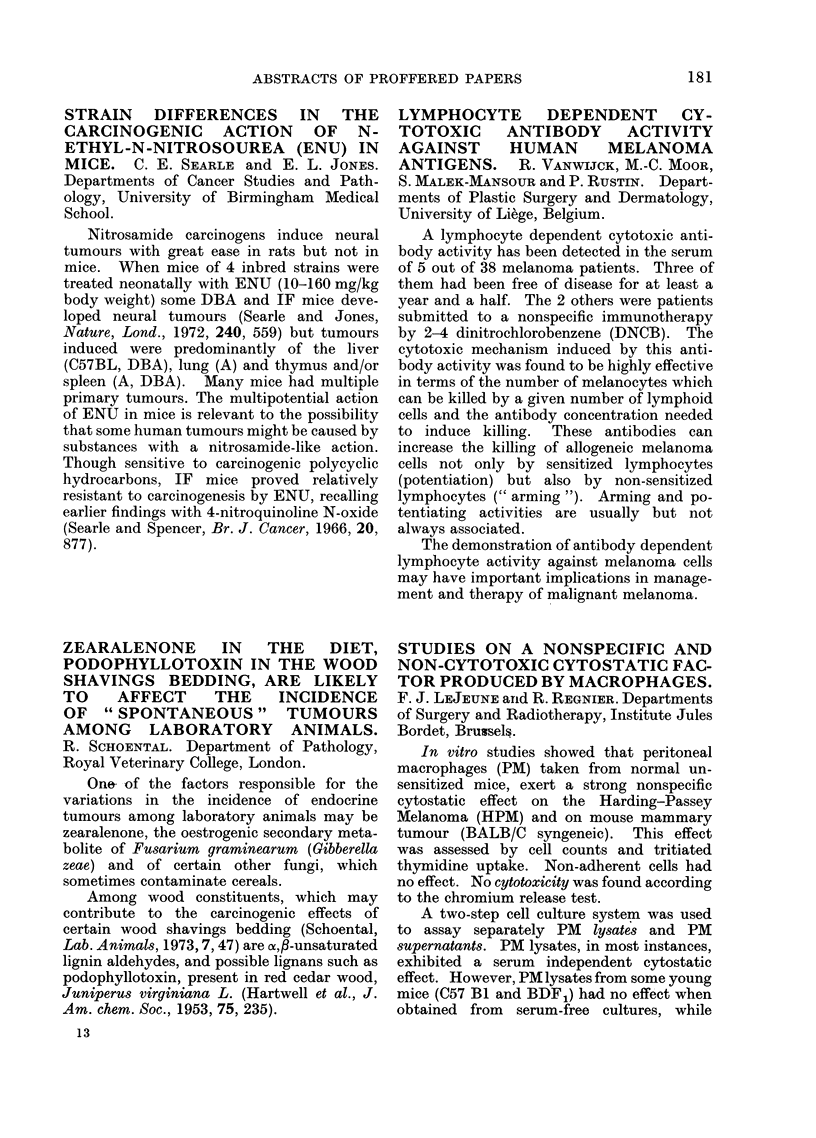# Proceedings: Lymphocyte dependent cytotoxic antibody activity against human melanoma antigens.

**DOI:** 10.1038/bjc.1974.160

**Published:** 1974-08

**Authors:** R. Vanwijck, M. C. Moor, S. Malek-Mansour, P. Rustin


**LYMPHOCYTE DEPENDENT CYTOTOXIC ANTIBODY ACTIVITY AGAINST HUMAN MELANOMA ANTIGENS.** R. VANWIJCK, M.-C. MOOR, S. MALEK-MANSOUR and P. RUSTIN. Departments of Plastic Surgery and Dermatology, University of Liège, Belgium.

A lymphocyte dependent cytotoxic antibody activity has been detected in the serum of 5 out of 38 melanoma patients. Three of them had been free of disease for at least a year and a half. The 2 others were patients submitted to a nonspecific immunotherapy by 2–4 dinitrochlorobenzene (DNCB). The cytotoxic mechanism induced by this antibody activity was found to be highly effective in terms of the number of melanocytes which can be killed by a given number of lymphoid cells and the antibody concentration needed to induce killing. These antibodies can increase the killing of allogeneic melanoma cells not only by sensitized lymphocytes (potentiation) but also by non-sensitized lymphocytes (" arming "). Arming and potentiating activities are usually but not always associated.

The demonstration of antibody dependent lymphocyte activity against melanoma cells may have important implications in management and therapy of malignant melanoma.